# Phenotypic and Functional Heterogeneity of Bovine Blood Monocytes

**DOI:** 10.1371/journal.pone.0071502

**Published:** 2013-08-14

**Authors:** Jamal Hussen, Anna Düvel, Olivier Sandra, David Smith, Iain Martin Sheldon, Peter Zieger, Hans-Joachim Schuberth

**Affiliations:** 1 Immunology Unit, University of Veterinary Medicine, Hannover, Germany; 2 UMR1198 Biologie du Développement et Reproduction, Institut National de la Recherche Agronomique, Jouy-en-Josas, France; 3 Ecole Nationale Vétérinaire d’Alfort, Maisons-Alfort, France; 4 Institute of Infection, Immunity and Inflammation, College of Medical, Veterinary and Life Sciences, University of Glasgow, Glasgow, United Kingdom; 5 Institute of Life Science, School of Medicine, Swansea University, Swansea, United Kingdom; 6 Zoetis, Paris, France; The Ohio State University, United States of America

## Abstract

Murine and human peripheral blood monocytes are heterogeneous in size, granularity, nuclear morphology, phenotype and function. Whether and how bovine blood monocytes follow this pattern was analyzed in this study. Flow cytometrically, classical monocytes (cM) CD14^+^ CD16^−^, intermediate monocytes (intM) CD14^+^ CD16^+^ and nonclassical monocytes (ncM) CD14^+^ CD16^+^ were identified, with cM being the predominant subset (89%). cM showed a significant lower expression of CD172a, intM expressed the highest level of MHC class II molecules, and ncM were low positive for CD163. Compared to cM and intM, ncM showed a significantly reduced phagocytosis capacity, a significantly reduced generation of reactive oxygen species, and reduced mRNA expression of CXCL8, CXCL1 and IL-1β after LPS stimulation. Based on IL-1β secretion after LPS/ATP stimulation, the inflammasome could be activated in cM and intM, but not in ncM. IFNγ increased the expression of CD16 selectively on cM and induced a shift from cM into intM in *vitro*. In summary, bovine CD172a-positive mononuclear cells define three monocyte subsets with distinct phenotypic and functional differences. Bovine cM and intM share homologies with their human counterparts, whereas bovine ncM are not inflammatory monocytes.

## Introduction

Monocytes are immune cells linking innate and adaptive immunity and play an essential role as cells of the first line of defense against pathogens [1]. Monocytes originate in the bone marrow and are released into the peripheral blood, where they circulate for several days before entering tissues to replenish tissue macrophage or dendritic cell populations [2]. Recent studies have reported a consecutive migration of murine monocyte subsets: In response to tissue damage or infection, nonclassical monocytes (ncM) rapidly migrate out of the circulation and invade the damaged site. This early efflux of ncM is transient and rapidly followed by the recruitment of neutrophils and subsequently by classical monocytes (cM) [3]. Monocytes are equipped with a large array of receptors that recognize various pathogens and mediate phagocytosis, and stimulated monocytes can produce large amounts of reactive oxygen species (ROS), chemokines and cytokines (e.g. chemokine (C-X-C motif) ligand 8 (CXCL8), CXCL1, interleukin (IL)-1β and tumor necrosis factor (TNF)-α) which are involved in the defense against pathogens [4], [5].

Studies in human and mice have shown that blood monocytes are a heterogeneous population and that monocyte subsets exert different functions. [6]. Two subpopulations of human monocytes (CD14^++^CD16^−^ and CD14^+^CD16^+^ monocytes) were initially defined based on the differential expression of the lipopolysaccharide (LPS) receptor CD14 and the FcγIIIR CD16 [7]. Subsequent studies identified considerable heterogeneity within the CD16-positive monocyte population and allowed for discrimination between CD14^++^CD16^+^ and CD14^+^CD16^++^ subsets [8]. Based on the new nomenclature, human monocytes are subdivided into three subsets on the basis of surface CD14 and CD16 expression. The major population of human monocytes (90%) with high CD14 but no CD16 expression (CD14^++^ CD16^−^) are now termed cM, whereas the minor population (10%) of human monocytes contain the intermediate monocytes (intM) subset, with low CD16 and high CD14 expression (CD14^++^ CD16^+^), and the ncM subset, with high CD16 and low CD14 expression (CD14^+^ CD16^++^) [9]. In the murine system classical monocytes (cM) are defined as Ly6C^++^CD43^+^, intermediate monocytes (intM) as Ly6C^++^CD43^++^ and nonclassical monocytes (ncM) as Ly6C^+^CD43^++^ [10]. Human cM have been shown to act as professional phagocytes, to generate ROS and to secrete cytokines in response to bacterial stimulation [9]. Recently, human intM were considered as the monocytes subpopulation with the highest inflammatory potential, being the main producers of ROS [11] and inflammatory cytokines [12]. Human and mouse ncM have been characterized as the monocyte subset which patrol and crawl along the endothelium in a leukocyte function-associated antigen (LFA)-1 dependent manner [12]. NcM have further been shown to sense the presence of viruses and nucleic acids via toll-like receptor (TLR)-7 and TLR-8 [12]. Some studies proposed that monocytes leave the bone marrow as classical cells, which can either directly invade inflamed tissues and differentiate into macrophages and/or dendritic cells, or they can differentiate into intermediate (CD14++CD16+) monocytes in the circulation [9], [13], [14].

Studies on bovine monocytes were so far restricted to the CD14-positive mononuclear cells (MNC) population [15], [16], [17], [18]. Therefore, this study aimed at the definition of bovine blood monocyte subsets and the characterization of their phenotypical and functional heterogeneity.

## Materials and Methods

### Ethics Statement

This study was approved by the Niedersächsisches Landesamt für Verbraucherschutz und Lebensmittelsicherheit (AZ 33.9-42502-05-09A/598). All procedures involving animals were carried out in accordance with German legislation on animal welfare.

### Separation of Mononuclear Cells

Blood from healthy nonlactating, pluriparous German Holstein cows was obtained by venipuncture of the vena jugularis externa into heparinized vacutainer tubes (Becton Dickinson, Heidelberg, Germany). The blood was layered on Ficoll-Isopaque (PAA, Pasching, Austria) and centrifuged at 4°C for 30 min at 1000×g. The interphase containing MNC was washed 3 times (10 min, 4°C) in PBS (500×g, 250×g, 100×g) and finally suspended in culture medium.

### Flow Cytometric Analysis of Monocyte Subsets

Surface marker expression on bovine monocyte subsets was determined using single and multiple labeling of bovine MNC (n = 6 animals) or MACS-separated monocyte subsets (n = 5 animals) using monoclonal antibodies to the molecules: CD16 (MCA5665F), CD14 (MCA1568PE), CD172a (MCA2041P647), MHC-II (MCA2226F), CD163 (MCA2311PE), CD4 (MCA1653A647), CD8 (MCA837A647), CD21 (MCA1424PE ) and CD335 (MCA2365PE) (all antibodies: AbD Serotec, Oxford, UK). Peripheral blood mononuclear cells (4×10^5^) or MACS-separated monocyte subsets (1×10^5^) from healthy animals were incubated with fluorochrome labeled monoclonal antibodies in PBS containing bovine serum albumin (5 g/L) and NaN_3_ (0.1 g/L). After 30 minutes in the dark (4C°), labeled cells were washed once and analyzed using the Accuri C6 flow cytometer (BD Biosciences). Dead cells were excluded from the analysis by adding propidium iodide (PI, 2 µg/ml, Calbiochem, Bad Soden, Germany). At least 100 000 total MNC or 20.000 monocytes were collected and analyzed with the CFlow Software, Version 1.0.264.15 (BD Biosciences).

### Isolation of Bovine Monocyte Subsets

Bovine monocyte subsets were isolated using a tow-step (multiSort) MACS procedure according to their different CD14 and CD16 expression. After density gradient separation, mononuclear cells were labeled with a FITC-conjugated CD16-specific antibody (AbD Serotec, Oxford, UK) for 15 min and then with anti-FITC MultiSort MicroBeads (Anti-FITC MultiSort Kit; Miltenyi Biotec) to separate MNC into a CD16-positive fraction (containing intM and ncM) and a CD16-negative fraction (containing cM and other blood MNC). Bound beads to the CD16-positive fraction were released by incubation with MultiSort release reagent (included in the Anti-FITC MultiSort Kit) for 10 min. The CD16-negative and the CD16-positive the cell fractions were subsequently incubated with anti-CD14 MicroBeads (Miltenyi Biotec) and separated according to the manufacturer’s instructions: CD14-positive cells were positively selected from the CD16-negative fraction (cM) and from the CD16-positive fraction (intM). Flow through cells of the CD16-positive fraction incubated with anti-CD14 MicroBeads presented ncM. All steps of monocyte subset isolation were performed at 4°C. After each labeling step, the cells were washed with PBS-EDTA (300×g, 10 min). Negatively and positively selected cells were checked flow cytometrically for their viability and purity after labeling the cells with a PE-conjugated mouse anti bovine CD14 monoclonal antibody (ABD Serotec) and adding propidium iodide (2 µg/ml). This separation procedure yielded a purity of 94.9% ±5.4% for cM, 82.9% ±4.5% for intM and 90.3% ±2.2% for ncM. The mean viability was 92% ±5% for all subsets (n = 5 animals).

### Generation of ROS

ROS generation was performed in 96-well round-bottom microtiter plates (Corning, NY, USA). Bovine MNC (3×10^5^/well) in RPMI medium were incubated with heat killed and serum opsonized (45 min incubation with heat inactivated bovine serum; 37°C, 5% CO_2_ and subsequently washed with RPMI medium) *Escherichia coli* (*E. coli*) (50 bacteria/cell) or with 50 nmol/l PMA (Sigma, Germany) for 15 min (37°C, 5% CO_2_). For the detection of ROS, dihydrorhodamine (DHR 123, Mobitec, Goettingen, Germany) was added to the cells (750 ng/ml final). To identify monocyte subsets, combinations of fluorochrome-conjugated monoclonal antibodies to CD172a (MCA2041P647), CD14 (MCA1568A647) and MHC-II (MCA5655PE) were added to the cells. After incubation the cells were washed with RPMI medium and suspended in medium containing propidium iodide (PI, 2 µg/ml final, Calbiochem, Bad Soden, Germany) to exclude dead cells from the analysis. Using a combination of CD172a- and CD14-specific monoclonal antibodies, ncM were identified as CD172a-positive and CD14-negative. Using a combination of CD14- and MHC-II-specific monoclonal antibodies, intM and cM were identified as both CD14-positive and MHC-II-positive cells with a higher MHC-II expression (MHC-II^++^) on intM. The relative amount of generated ROS was determined by the median green fluorescence intensity of gated monocyte subsets after acquisition of 20 000 events (n = 5 animals).

### Phagocytosis Assay

Heat killed *staphylococcus aureus* (*S. aureus*) (Pansorbin, Calbiochem, Merck, Nottingham, UK) or *E. coli* (Institute of Microbiology, University of Veterinary Medicine, Hannover, Germany) were labeled with fluoresceinisothiocyanate (FITC, Sigma-Aldrich, St. Louis, Missouri, USA). FITC-conjugated and heat killed *S. aureus* or *E. coli* were opsonized with heterologous bovine serum (diluted 1∶10 with PBS) for 45 minutes at 37°C. Finally, bacteria were centrifuged at 14,000×g for 5 min and Pellet was suspended in RPMI medium and adjusted to 2×10^8^ bacteria/mL after counting the bioparticles flow cytometrically. MACS-separated bovine monocyte subsets were plated in 48 well plates (1×10^5^/well) and incubated for 24 h (37°C, 5% CO_2_) in RPMI medium. After this resting period, cells were incubated with opsonized bacteria (50 bacteria/cell) for 40 minutes (37°C, 5% CO_2_). Control samples were incubated without bacteria. After incubation, samples were flow cytometrically analyzed after addition of propidium iodide (PI, 2 µg/ml final) to exclude dead cells. Phagocytic activity of the three monocyte subsets was defined as the percentage of green fluorescing cells among viable cells (n = 4 animals).

### *In vitro* Stimulation of Monocyte Subsets and RT-qPCR

MACS-separated monocyte subsets were suspended in RPMI medium (PAA, Pasching, Austria) supplemented with 10% (v/v) fetal calf serum and penicillin/streptomycin at 2×10^5^ cells per ml. The cells were cultivated in 24 well plates (Costar, Cambridge, MA) at 2×10^5^ cells per well for 24 h (37°C, 5% CO_2_). After this resting period, cells were stimulated for 3 h with LPS (1 µg/ml) (37°C, 5% CO_2_). Total RNA of the cells was extracted for quantitative real-time PCR analysis (see below) of selected cytokine and chemokine mRNA expression. RNA preparation and RT-qPCR has been described recently [19]. In brief: Extraction of RNA was performed using the RNeasy Plus Micro Kit (Qiagen, Hilden, Germany) including the elimination of contaminating genomic DNA with a DNA eliminator column. Integrity and quality based on 18s- and 28s- units of RNA was checked by a microfluidic chip-based automated electrophoresis system (Experion Automated Electrophoresis System, Biorad, Munich, Germany). Experion RNA HighSense Chips were used according to the manufacturer’s instructions. With a total-RNA amount of 8 ng cDNA synthesis was performed using the Superscript++Reverse Transcriptase and oligo (dT) nucleotide primers (both Invitrogen, Karlsruhe, Germany) according to the manufacturer’s instructions. Subsequent RT-qPCR was conducted using the Power SYBR Green PCR Master Mix (Applied Biosystems, Darmstadt, Germany). Primers for the analyzed genes are listed in Table 1. Thermocycling was performed in a StepOnePlus Real Time PCR system (Applied Biosystems, Darmstadt, Germany). The quality of amplification was verified by melt curve analysis. A dilution series (10^6^–10^2^ copies) of cDNA subclones was analyzed for each gene simultaneously with the samples and used for the determination of the relative copy numbers of individual transcripts. The amplification efficiency was calculated from the slope of the standard curve by the formula: E = 10^−1/slope^ for each run and ranged between 90% and 110% (Cells from 3 different animals, samples were tested in duplicates in the RT-PCR).

**Table 1:** Primer sequences and concentrations used for real-time PCR analysis.

Gene	Forward (for) and reverse (rev) primer sequences (5′ ->3′)and concentrations (nM)	bp	Reference
IL1B	for TTC TCT CCA GCC AAC CTT CAT T (300)	198	[32]
	rev ATC TGC AGC TGG ATG TTT CCA T (300)		
TNF	for CTT CTG CCT GCT GCA CTT CG (300)	156	[33]
	rev GAG TTG ATG TCG GCT ACA ACG (300)		
CXCL8	for CCTCTTGTTCAATATGACTTCCA (900)	170	[33]
	rev GGCCCACTCTCAATAACTCTC (50)		
CXCL1	for CGCCTGTGGTCAACGAACT (300)	83	[34]
	rev CACCTTCACGCTCTGGATGTT (300)		
iNOS	for GAGATAGAAACAACAGGAACCTAC (300)	70	mod.^a^ [35])
	rev AGGCCTGCTTGGTGGCGAA (50)		
ArgI	for ATCTGGGTTGATGCCCATGC (300)		Novel design, Acc. no^b^.:
	rev AGGAAAATCCTGGGAGCTGT (50)	100	NM_001017942

a mod., modified.

b Acc.no., Gene Bank accession number of nucleotide sequence used for primer generation.

### *In vitro* Stimulation of IL-1β Secretion

MACS-separated monocyte subsets (n = 5 animals) were suspended in IMDM medium (PAA, Pasching, Austria) supplemented with 10% (v/v) fetal calf serum and penicillin/streptomycin at 1×10^5^ cells per ml. The cells were cultivated in 24 well plates (Costar, Cambridge, MA) at 1×10^5^ cells per well for 24 h (37°C, 5% CO_2_). After this resting period, cells were incubated with either culture medium (IMDM, negative control) or LPS (1 µg/mL, Sigma-Aldrich, Germany) for 4 h (37°C, 5% CO_2_). Adenosine triphosphate (ATP) (5 mmol/L) was added to the culture for an additional hour. After a total of 5 hours in *vitro*, cell culture supernatants were collected and stored at −70°C until assayed [20].

### IL-1β ELISA

Flat-bottomed 96-well plates (MaxiSorp; Nunc, Roskilde, Denmark) were coated with monoclonal anti-ovine IL-1β (AbD Serotec, 100 µL/well, 4 µg/mL) in carbonate-bicarbonate buffer (15 mmol/l Na_2_CO_3_, 35 mmol/l NaHCO_3_, pH 9.6) and incubated for 18 h at 4°C. Plates were washed five times with phosphate buffer (2.5 mmol/l NaH_2_PO_4_, 7.5 mmol/l Na_2_HPO_4_, 145 mmol/l NaCl, 0.1% (v/v) Tween 20, pH 7.2). Each well was filled with 75 µl of sample and plates were incubated on a rotating platform for 1 hour at RT. Serial dilutions of purified bovine recombinant IL-1β (AbD Serotec) were included in each assay to construct a standard curve. Samples and recombinant IL-1β were tested in duplicates. Plates were washed five times with phosphate buffer and wells were filled with 75 µl of rabbit anti-bovine IL-1β polyclonal antibody (AbD Serotec, 2 µg/mL). Plates were incubated for 1 h at room temperature, washed, and peroxidase-conjugated AffiniPure goat anti rabbit IgG (FC) (Dianova) was added. After 1 h at RT the plates were washed and subsequently filled with 75 µl/well peroxidase substrate buffer (33.3 mmol/l citric acid, 66.7 mmol/l NaH_2_PO_4_, pH 5.0) supplemented with 130 µg/mL 3,3′,5,5′-tetramethylbenzidine (TMB, Sigma, Germany) and 0.01% (v/v) H_2_O_2_ (all chemicals from Sigma-Aldrich, Germany). After 20 min at RT in the dark adding 75 µl of 0.5 M H_2_SO_4_ to each well stopped the reaction. Optical densities were read in an ELISA microplate reader (MR 5000, Dynatech, Denkendorf, Germany) at 450 nm and 630 nm. Bovine IL-1β concentrations were calculated by referring to a standard curve. The detection limit of the assay was 100 pg/mL.

### Whole Blood Stimulation

Blood from healthy cattle (n = 7 animals) was incubated with recombinant bovine interferon (IFN)γ (10 ng/ml), IL-4 and IL-13 (5 ng/ml of each), CCL-5 (50 nmol/l), TNF-α (10 ng/ml) or IL-1β (10 ng/ml) for 4 h (37°C, 5% CO_2_). After incubation the blood was diluted with phosphate buffer saline (1∶1) and centrifuged at 4°C for 10 min at 1000×g and the supernatant fluid was expelled. Twice hypotonic lysis of erythrocytes was done by adding distilled water (20 ml for 20 sec.) followed by the addition of 20 ml double concentrated PBS. Finally the cells were suspended in PBS and stained with monoclonal antibodies to CD16 (MCA5665F), CD14 (MCA1568PE) and CD172a (MCA2041P647) (see above).

### Statistical Analyses

Statistical analysis was carried out using the software Prism (GraphPad software). Results are expressed as means ± S.E. of the mean (SEM). Differences between means were tested with one-factorial analysis of variance (ANOVA) and Bonferroni’s correction for normally distributed data. Results were considered statistically significant at a p-value of less than 0.05.

## Results

### Phenotypic Characterization of Bovine Monocyte Subsets

**Figure 1:**
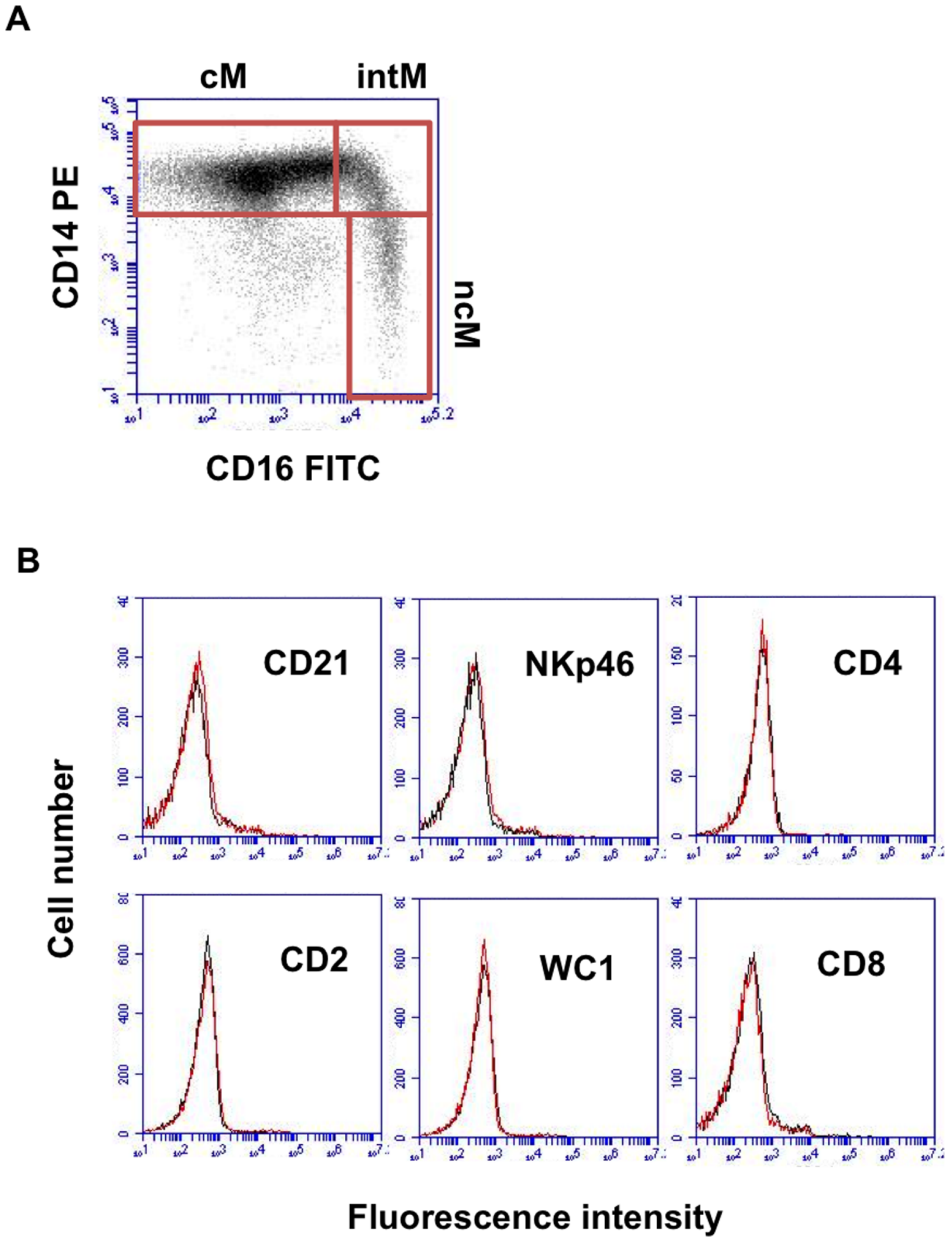
Gating strategy of bovine monocyte subsets based on relative CD14 and CD16 expression. (**A**) Three-color immunofluorescence of bovine MNC with mAbs to CD172a, CD14 and CD16 defines three monocyte subsets in peripheral blood. Viable (propidium-iodide-negative) mononuclear cells, based on forward and side scatter characteristics, were gated on CD172a-positive cells. Dot plots of CD14 and CD16 expression display classical monocytes (CD14+CD16−, upper left), intermediate monocytes (CD14+CD16+, upper right) and nonclassical monocytes (CD14-CD16+, lower right). (**B**) Monocytes were gated in a side scatter/CD172a dot plot, identifying monocytes as CD172a-positive cells. Histograms show that CD172a-positive cells do not express common markers of B cell (CD21), T cell (CD2, CD4 or CD8), NK cell (NKp46) or blood γδ T cell (WC1). Data are shown for cells from one of five tested animals.

**Figure 2:**
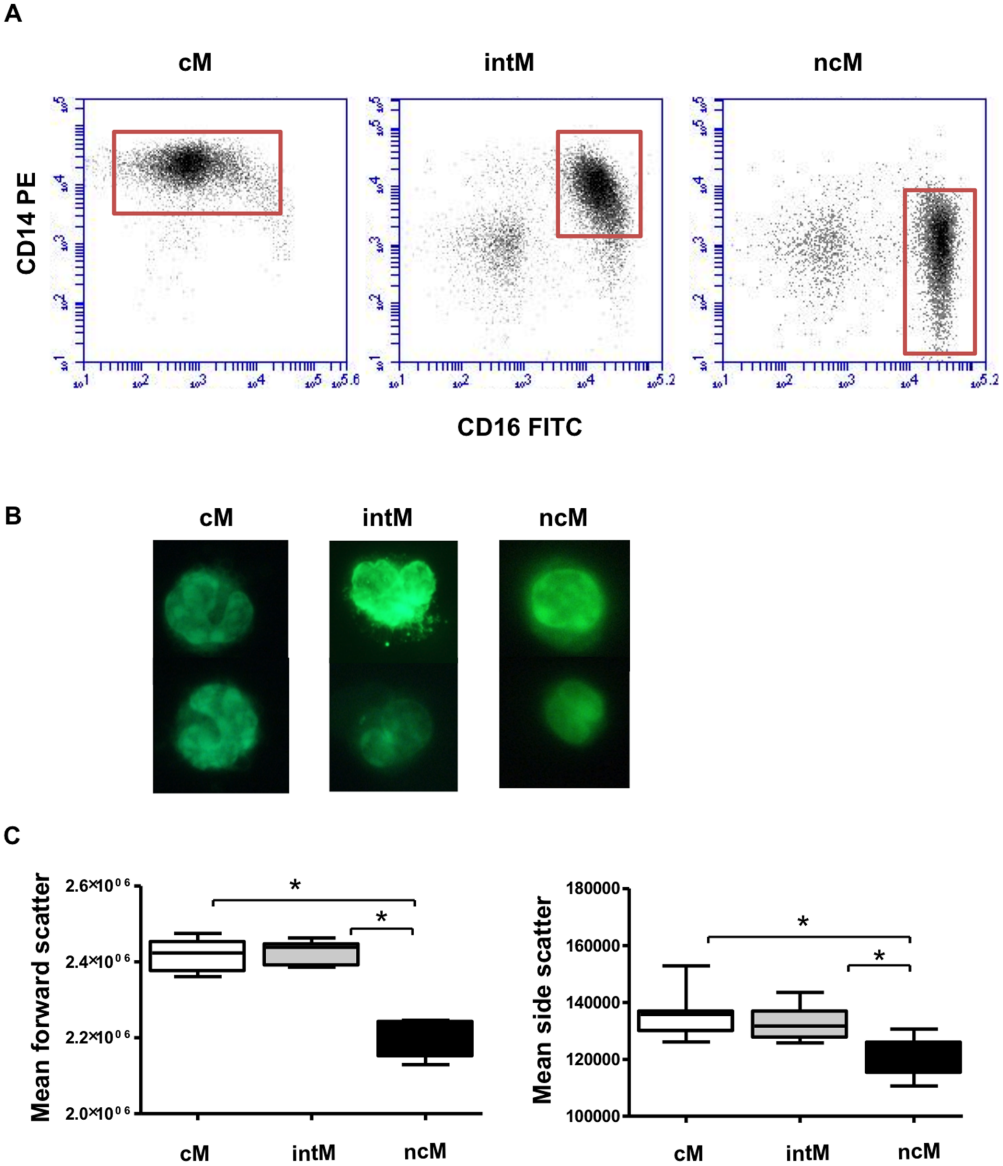
Purification of bovine monocyte subsets. (**A**) Bovine cM (CD14^+^ CD16^−^), intM (CD14^+^ CD16^+^) and ncM (CD14^−^ CD16^+^) subsets were separated from bovine MNC using a two-step MACS procedure as described in material and methods. Separated subsets were labeled with monoclonal antibodies to CD14 and CD16 to identify their purity. Representative examples from 5 independent experiments are shown. (**B**) Microscopic images of separated monocyte subsets are shown with magnification 100 after staining with acridine orange. (**C**) Bovine MNC were labeled with monoclonal antibodies to CD14 and CD16 to identify three monocyte subsets. Gated monocyte subsets were presented in FSC against SSC. The median of FSC and SSC of the individual subsets was measured (n = 6 cows) and presented as mean ± SEM. Statistical analysis of significance were performed using ANOVA and Bonferroni’s correction for normally distributed data. *(P<0,01).

**Figure 3:**
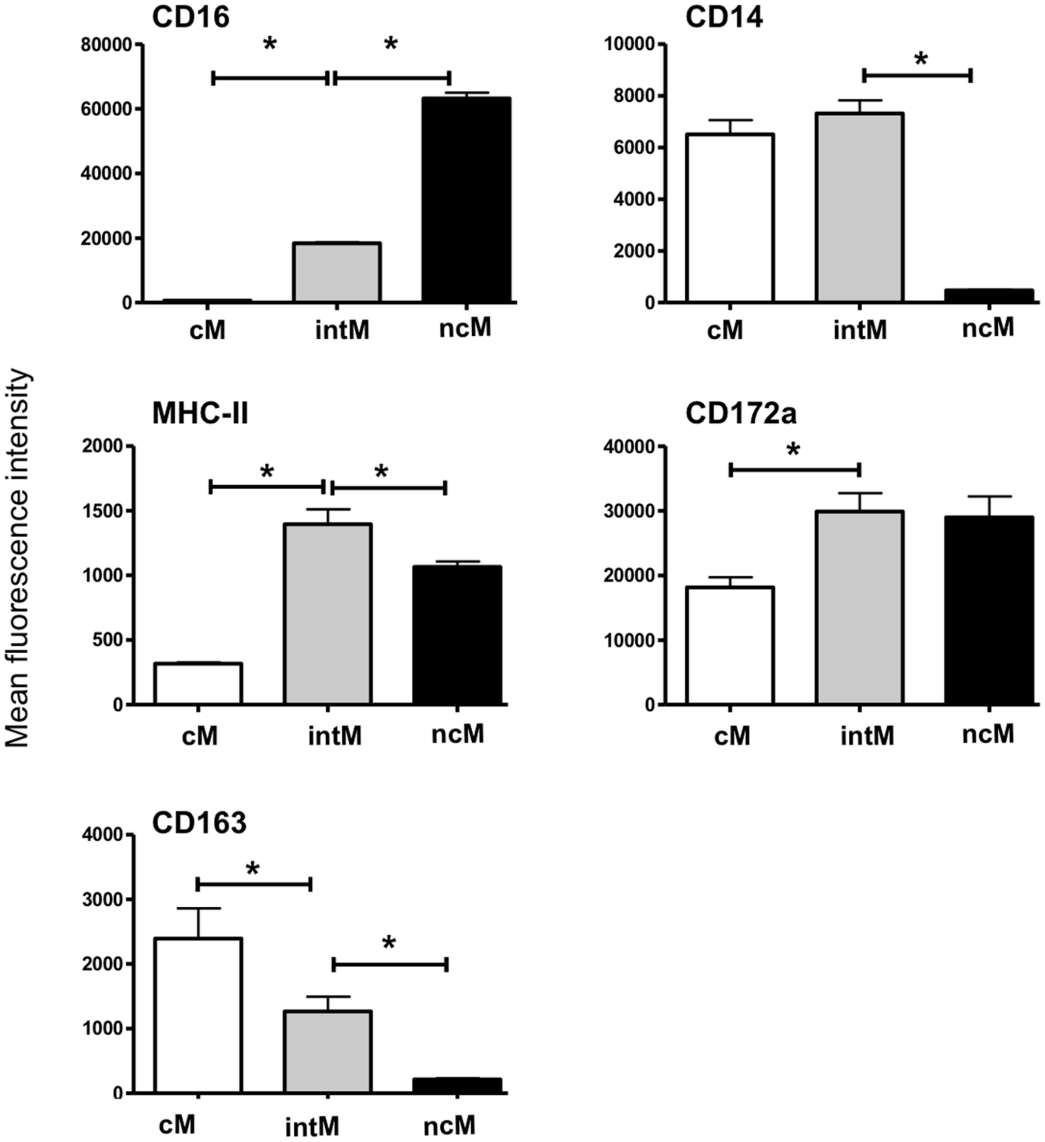
Flow cytometric analysis of established monocyte surface markers. MACS-separated monocyte subsets (n = 5 cows) were labeled with antibodies to different monocyte markers or isotype controls. After dead cell exclusion with propidium iodide, data were measured as median fluorescence intensity (MFI) and presented as means ± SEM. Background fluorescence (measured in negative controls) was subtracted. Statistical analysis of significance was performed using ANOVA and Bonferroni’s correction for normally distributed data *(P<0,01).

Bovine monocytes were identified based on CD172a expression. CD172a-positive cells were negative for the T cell marker CD4 and CD8, the B cell marker CD21, the NK cell marker NKp46 (CD335), and the γδ T cell marker WC1 (Fig. 1B). Based on the cell surface expression of CD14 and CD16, three blood monocyte subsets could be defined after flow cytometry (Fig. 1A, Table 2). The major subpopulation of bovine monocytes (cM) showed high expression of CD14 and no expression of CD16 (CD14^+^/CD16^−^). A second subset of monocytes (intM) expressed highly CD14 and displayed low expression of CD16 (CD14^+^/CD16^+^). A third monocyte subset was defined by high expression of CD16 and no expression of CD14 (CD14^−/^CD16^+^). After magnetic-activated cell sorting of three monocyte subsets (Fig. 2A), acridine orange stained nuclei appeared to be generally reniform. NcM appeared to be slightly smaller (Fig. 2B) which was proven by flow cytometry: ncM displayed significantly lower FSC (p<0.001) and SSC (p<0.05) values compared to classical and intermediate monocytes (Fig. 2C). Monocyte subsets differed significantly in their expression of CD163, CD172a, and MHC class II molecules. The expression level of CD172a was higher on intM and ncM, whereas the CD163 was expressed highest on cM. IntM showed the highest MHC class II expression (Fig. 3). Sorted monocyte subsets were negative for the T cell marker CD4 and CD8, the B cell marker CD21, the NK cell marker NKp46 (CD335), or WC1 (a marker of a subset of γδ T cells) (Data not shown).

**Table 2:** Fractions of monocyte subsets of MNC or CD172a+ monocytes and cell numbers in bovine peripheral blood.

	cM	intM	ncM
% of MNC	18±3	0.9±0.3	1.3±0.4
% of total monocytes	89±3	4.4±1.0	5.7±1.3
Cells/ml blood (x 10^4^)	84±10	4.0±0.9	5.8±0.9

N = 5 cows. cM: classical monocytes; intM intermediate monocytes; ncM nonclassical monocytes.

### Monocyte Subsets Differ in their Capacity to Phagocytize and to Generate Reactive Oxygen Species

**Figure 4:**
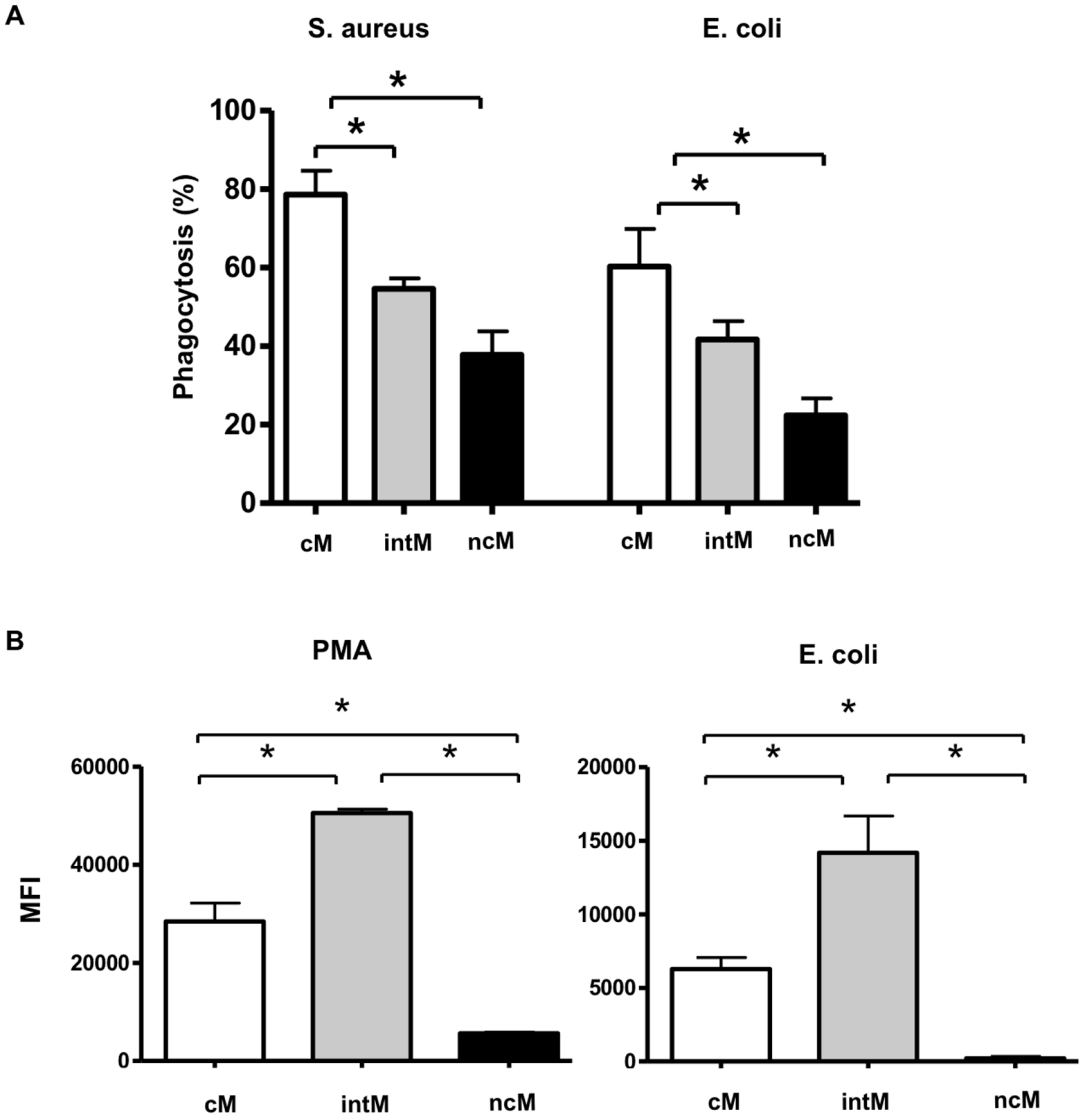
Phagocytosis and ROS-production capacity of bovine monocyte subsets. (**A**) MACS-separated cM, intM and ncM subsets (1×105/well) were incubated with heat killed FITC-labeled *S. aureus* or *E. coli*, which have been opsonized with bovine serum for 45 minutes. Control cells were incubated only in medium. The percentage of FITC-positive cells within viable (PI-negative) cells were determined flow cytometrically for the three monocyte subsets and are denoted as mean value ± SEM (n = 3 animals). (**B**) Flow cytometric analysis of ROS-formation by bovine monocyte subsets. Bovine MNC (n = 4 animals) were stimulated with PMA or heat killed and serum opsonized *E. coli* in the presence of DHR-123. The ncM subset was identified after labeling the cells with antibodies to CD172a and CD14 as CD172a positive and CD14 low cells. In a combination of CD14 and MHC-II monoclonal antibodies, the intM and cM subsets could be identified as both CD14 high cells with higher MHC-II expression on the intM subset. Cells were gated on viable (propidium-iodide-negative) CD172a-positive cells or CD14 high cells. ROS generation was calculated as the median green fluorescence intensity of gated monocyte subsets after subtracting the MFI of non-stimulated cells and presented as mean ± SEM. Differences between groups were calculated using the one-way ANOVA and were considered significant (*) if p<0.05.

To see whether monocyte subsets show differences in their antimicrobial functions, we analyzed the ability of the three subsets to phagocytize bacteria and to produce ROS. CM significantly phagocytized more serum-opsonized bacteria compared to intM and ncM (Fig. 4A). No significant difference between the capacity to phagocytize *S. aureus* or *E. coli* bacteria was observed. Whereas the low phagocytosis capacity of ncM corresponded to the nearly absent ability to generate ROS (Fig. 4B), intermediate monocytes displayed the highest ROS generation capacity. Whether ROS generation was induced by PMA or fixed *E. coli* bacteria had no influence on the monocyte subset capacity (intM>cM>ncM).

### Cytokine and Chemokine Expression Pattern in Bovine Monocyte Subsets

**Figure 5:**
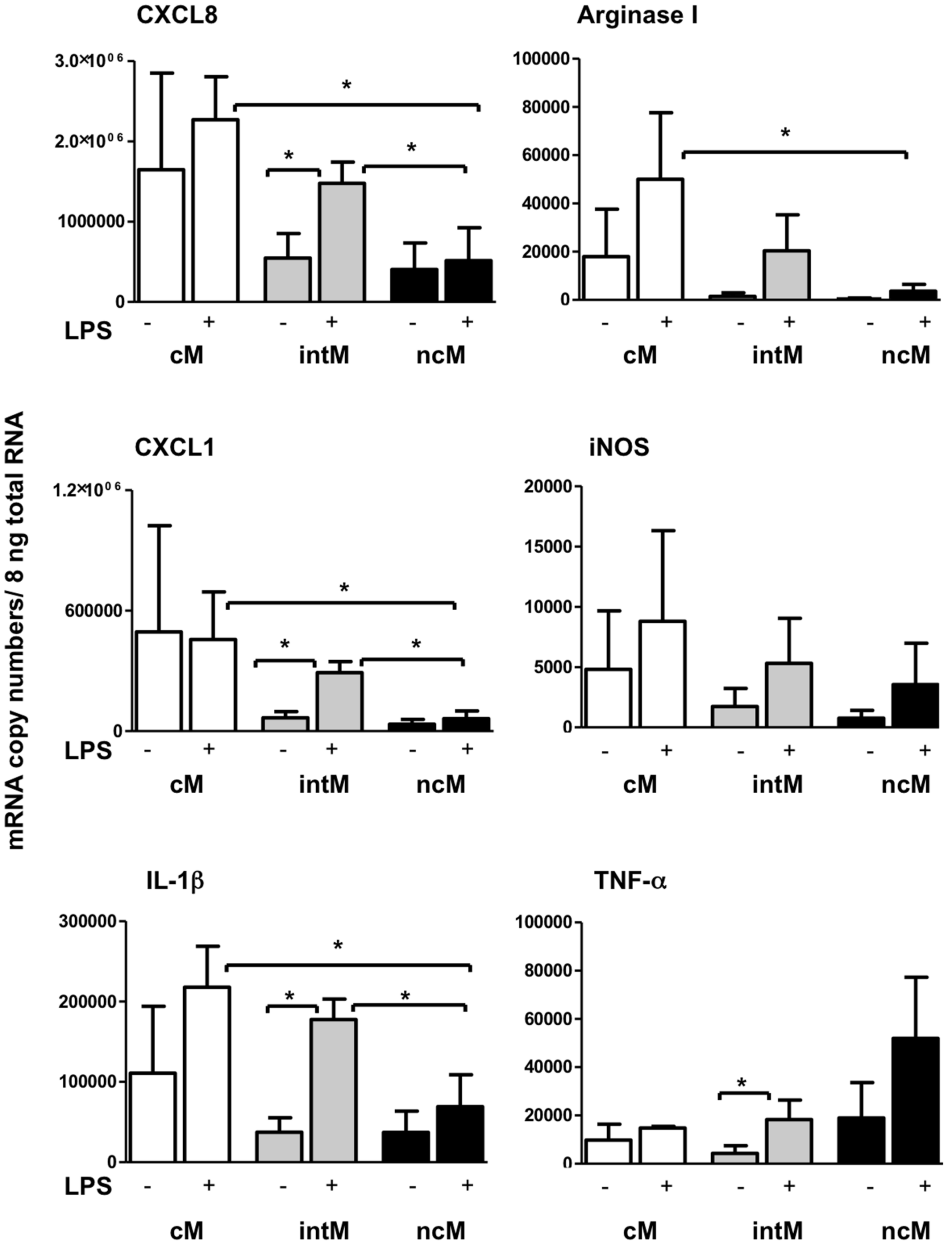
Chemokine and cytokine mRNA expression of bovine monocyte subsets. Bovine MACS-separated cM, intM and ncM (n = 3 animals) were stimulated with LPS (1 µg/ml) for 3 h. Messenger RNA copy numbers of the chemokines CXCL1 and CXCL8 and the cytokines IL-1β, Arginase1, iNOS and TNF-α were determined in 8 ng total RNA by quantitative RT-PCR. Samples were tested in duplicates. Differences between treatments and between groups (one-way ANOVA) were considered significant (*) at p<0.05.

To see whether the monocyte subsets are biased in their stimulated gene expression, we analyzed genes, which cover a spectrum of pro-inflammatory (IL1B, TNF, iNOS) and anti-inflammatory (ARG1) cytokines. Since monocytes are among the cells attracting neutrophils, two chemokines were analyzed which bind differentially to CXCR1 and/or CXCR2 on neutrophils. This would help us to understand the role of these subsets in the chemotaxis and functional modulation of neutrophils. IntM and ncM, displayed a homogenous baseline expression of the analysed genes, whereas cM showed up with highly variable baseline expression (Fig 5). Differences in baseline expression showed no statistical significance between the three monocyte subsets. For all analysed genes, LPS stimulation did not induce a significant upregulation in cM or ncM. In intM, LPS stimulation induced a significant (p<0.05) upregulation of CXCL8, CXCL1, IL1B, and TNF.

Among LPS-stimulated monocyte subsets, ncM showed significantly lower expression of CXCL8, CXCL1 and IL-1β compared to cM and intM, and a lower expression of Arginase I compared to cM (p<0.05).

### Inflammasome Activation in Bovine Monocyte Subsets

**Figure 6:**
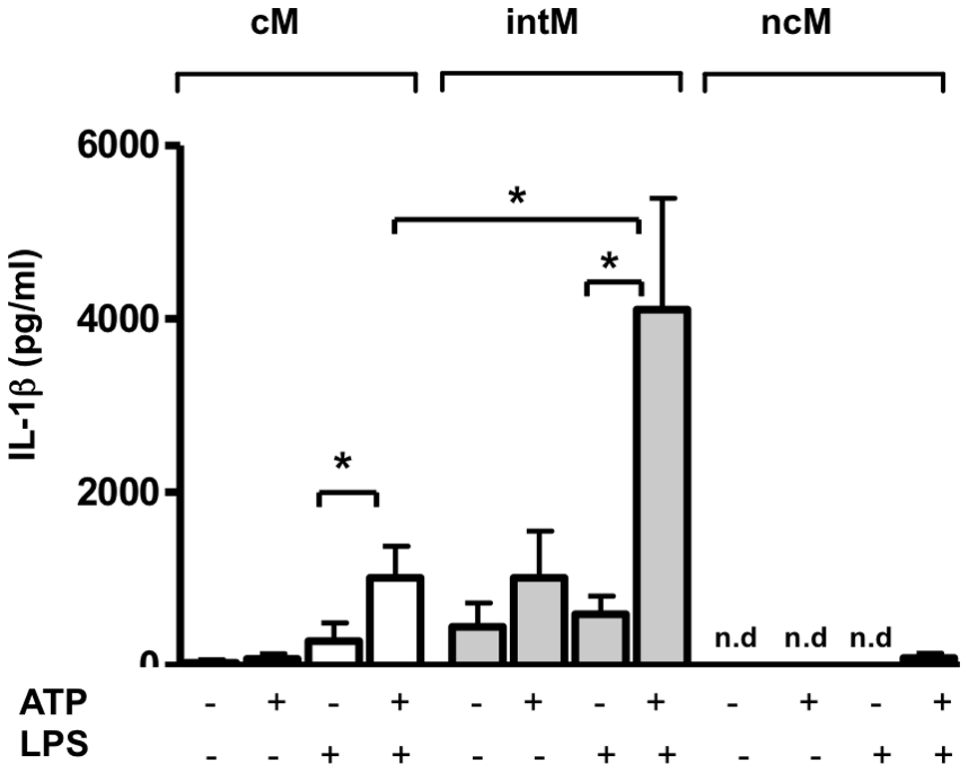
Inflammasome activation in bovine monocyte subsets. Analysis of inflammasome activation was done by measuring the secreted IL-1β in medium supernatants of monocyte subsets after combined stimulation with LPS and ATP. MACS-separated bovine monocyte subsets (n = 5 animals) were primed for 5 h with LPS (1 µg/mL) and 5 mmol/L ATP was added to the culture for an additional hour. IL-1β in cell supernatants was measured by ELISA. Differences between groups (one-way ANOVA) were considered significant (*) at p<0.05.

The secretion of the pro-inflammatory cytokine IL-1β requires two stimulating signals that activate the inflammasome. ATP has been shown to act as a second signal for the assembly of the NLRP3 inflammasome in LPS-primed bovine monocytes leading to the secretion of IL-1β [20]. IL-1β was determined in supernatants of LPS-primed/ATP-stimulated bovine monocyte subsets. The combined LPS/ATP stimulation of cM and intM resulted in a significant (12–13 fold) IL-1β increase versus stimulation with LPS as a single stimulus (Fig. 6). Supernatants of ncM, irrespective of activation, contained nearly no detectable IL-1β. Only the ncM of one animal showed a minimal secretion after treatment with LPS/ATP. Supernatant of intM stimulated with the combination of LPS/ATP contained significantly more IL-1β than those of LPS/ATP-stimulated cM.

### IFNγ Increases the Expression of CD16 on cM Monocytes

**Figure 7:**
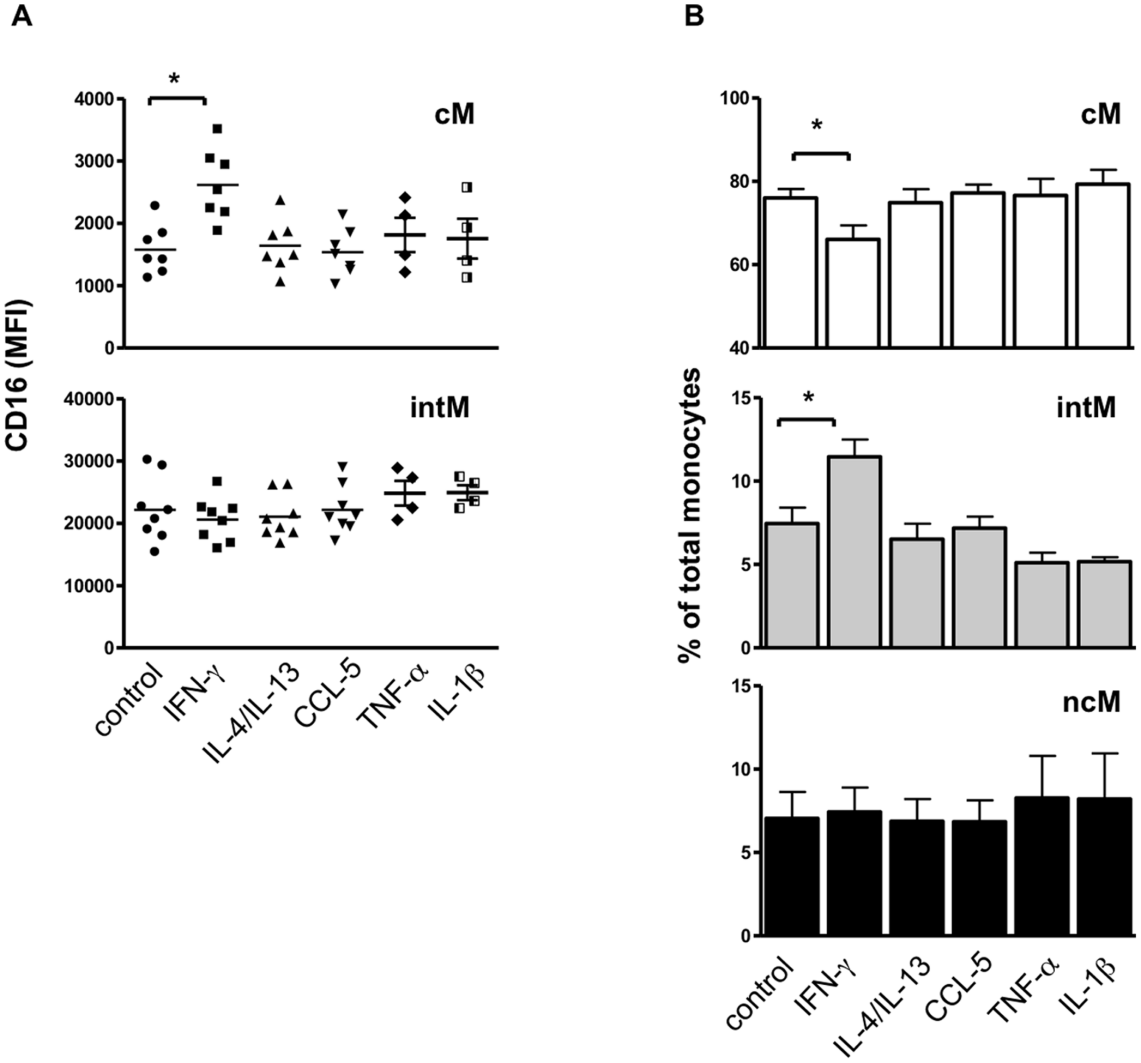
The effect of whole blood stimulation with different cytokines or CCL5 on monocyte subsets. Whole blood samples (n = 7 animals) were incubated with the cytokines IFNγ, IL-4/IL-13, TNF-α or IL-1β or the chemokine CCL5 for 4 h. Leukocytes were stained with monoclonal antibodies to CD172a, CD14 and CD16. After gating on vital (PI-negative) MNC, agate was made on CD172a-positive cells. CD172a-positive cells were presented in CD14 and CD16 dot plot. Mean fluorescence intensity of CD16 (A) and the percentage (B) of the three monocyte subsets was calculated and presented as mean ± SEM. Differences between groups were calculated using the one-way ANOVA and were considered significant (*) if p<0.05.

To test the hypothesis that a developmental relationship exists between monocyte subsets, whole blood was stimulated with inflammatory (IFNγ, IL-1β, TNF-α) and anti-inflammatory cytokines (IL-4, IL-13) as well as a monocyte attracting chemokine (CCL5). The stimulation with IFNγ selectively induced an upregulated expression of CD16 on classical monocytes (Fig. 7A). This shift significantly increased the percentage of intM among all monocytes and reduced the fraction of cM (Fig. 7B).

## Discussion

Studies in mice and human have shown that peripheral blood monocytes represent a heterogeneous population of circulating cells with differences in size, granularity, phenotype and function [9]. Since no data were available for bovine monocytes, this study aimed at the characterization of bovine peripheral blood monocyte heterogeneity.

Since bovine monocytes are usually described as CD14-positive [15], [16], [17], and one of the human monocyte subpopulations express very little or no CD14, we first identified a marker expressed on all putative bovine monocytes. An antibody specific for the Colony-Stimulating Factor-1 receptor (CD115) which is used to detect human and murine monocytes [2] was not available for bovine monocytes. Double staining of MNC with anti-CD172a, also known as signal-regulatory protein alpha (SIRPalpha), and antibodies specific for Nkp-46, CD2, CD4, CD8 or CD21 proved, that CD172a-positive cells among MNC are distinct from NK cells, T cells, and B cells. SIRPs have been shown to be expressed at high levels in human myeloid cells, including macrophages, monocytes, granulocytes and DC [21]. CD172a has been identified as pan monocyte marker of porcine monocytes [22], [23]. CD172a is also expressed at low levels on bovine [24] and porcine [25] plasmacytoid DC. Although not formally proven, CD172a-positive bovine monocyte subsets (defined by CD172a, CD14 and CD16 staining) are also distinct from blood dendritic cells, since they are negative for both CD14 and CD16 [2]. Thus, the use of CD172a as a marker to detect all monocytes in bovine peripheral blood is far superior over the use of CD14.

The recently updated classification of human monocyte heterogeneity identifies three human monocyte subsets, based on the differential expression of CD14 and CD16 [9]. This could also be shown for bovine monocytes after triple staining with antibodies specific for CD172a, CD14, and CD16. As in human blood [26], bovine cM constituted the majority (89.1%) of bovine peripheral blood monocytes. Relative expression densities of CD14, CD16, MHC-II and CD163 on bovine and human monocyte subsets also suggests a similar phenotype of monocyte subsets in both species [27]. This is also the case for the size of bovine and human ncM, being smaller and less granular compared to cM and intM [2]. Thus, regarding phenotype and frequency in blood, bovine monocytes appear to share many homologies with their human counterparts.

We next asked, whether the similarities also extend to the functionality of the monocyte subsets. We found the highest phagocytic capacity in bovine cM, which is in line with previously published data to the human cM subset [11], [12]. Bovine intM produced the highest amount of ROS and showed enhanced levels of inflammatory cytokines (TNF-α, IL-1β). This is comparable with newer studies describing human intM as main producers of ROS and pro-inflammatory cytokines [11], [12]. Overall, inflammatory responses, including phagocytosis, ROS generation and cytokine production in response to bacterial stimulation, appeared to be mediated by the two bovine CD14-positive monocyte subsets (cM, intM).

This is only partially overlapping with the human system, where the combined human CD16-positive subset (including intM and ncM) was generally termed ‘pro-inflammatory monocytes’ [28], [29]. Human CD16-positive monocytes produced high amounts of pro-inflammatory cytokines [30], [31] with human ncM producing the highest amount of IL-1β in response to LPS stimulation [27]. ROS generation of human ncM was equivalent to that of cM and intM [11].

In contrast, bovine ncM were nearly unable to respond to LPS/ATP stimulation with a detectable secretion of IL-1β. Moreover, they displayed the lowest phagocytic capacity of all bovine monocyte subsets, showed virtually no ROS generation and expressed lowest copy numbers of transcripts for all tested genes. Murine and human ncM [4], [12] crawl on endothelial cells and rapidly migrate into tissues in response to infection or tissue damage. This early efflux of ncM seems to be relevant for the subsequent recruitment of neutrophils [4]. The low mRNA expression of neutrophil-attracting chemokines in bovine ncM, which could not be stimulated by LPS, suggests a marginal role of these cells in the recruitment of neutrophils to the infected tissue. The possible function of bovine ncM in *vivo* is obscure.

Some studies proposed that intM are transitional cells bridging between cM and ncM, suggesting a developmental relationship between the monocyte subsets [9], [13], [14]. This may indeed be the case with bovine cells, since incubation of whole blood with INFγ induced an upregulated CD16 expression on cM causing an increased fraction of intM. This is in part supported by an earlier study in humans where the combined treatment of patients with M-CSF and IFNγ resulted in more CD16 expression on cM and the expansion of CD16-positive monocytes [14]. In our study we found no indications that IFNγ induces the differentiation of intM into ncM.

### Conclusions

Cell-surface expression of CD14 and CD16 among CD172a-positive cells define three functionally and phenotypically subsets of bovine monocytes resembling the human classical (CD14^+^CD16^−^), intermediate (CD14^+^CD16^+^), and nonclassical (CD14^+^CD16^+^) monocytes. Phenotypic and functional characteristics indicate similarities between bovine and human cM and intM, whereas significant functional differences exist between bovine and human ncM.
